# Patient-Reported Outcomes in Children Undergoing the Modified Green Procedure for Treating Sprengel’s Deformity: Results from a Multicentric Study

**DOI:** 10.3390/children12010018

**Published:** 2024-12-26

**Authors:** Giovanni Trisolino, Paola Zarantonello, Marco Todisco, Giovanni Luigi Di Gennaro, Grazia Chiara Menozzi, Philipp Scheider, Alessandro Depaoli, Diego Antonioli, Gino Rocca, Sebastian Farr

**Affiliations:** 1 Pediatric Orthopedics and Traumatology Unit, IRCCS Istituto Ortopedico Rizzoli, 40136 Bologna, Italy; marco.todisco@ior.it (M.T.); giovanniluigi.digennaro@ior.it (G.L.D.G.); graziachiara.menozzi@ior.it (G.C.M.); diego.antonioli@ior.it (D.A.); gino.rocca@ior.it (G.R.); 2 Orthopedics and Traumatology Unit, IRCCS Istituto Ortopedico Rizzoli, 44011 Argenta, Italy; paola.zarantonello@ior.it; 3 Department of Pediatric Orthopaedics and Adult Foot and Ankle Surgery, Orthopedic Hospital Speising, 1130 Vienna, Austria; philipp.scheider@oss.at; 4 Orthopedics and Traumatology Unit, IRCCS Istituto Ortopedico Bagheria, 90011 Bagheria, Italy; alessandro.depaoli@ior.it

**Keywords:** Sprengel, Sprengel’s deformity, shoulder, Green procedure, Green’s technique

## Abstract

Background: Sprengel’s Deformity (SD) is a rare condition of the shoulder girdle, appearing as the principal congenital anomaly of the shoulder in paediatric patients. The aim of this study is to document the combined experience of two paediatric orthopaedic departments in managing SD using the modified Green Procedure, with a specific emphasis on the clinical and functional outcomes reported by patients; Methods: from June 2010 to February 2023, 42 shoulders in 40 paediatric patients were surgically treated for SD at two paediatric orthopaedic departments. All patients were treated using the modified Green Procedure with or without clavicle osteotomy. To better evaluate the deformity, the Cavendish’s classification for aesthetic appearance and the Rigault’s classification for radiological aspect were used, while movements of abduction and flexion were quantified to assess shoulder mobility. Several dedicated questionnaires such as QuickDASH, the Shoulder Pain Index and the Shoulder Disability Index (SPADI) and finally the UCLA Shoulder Scale were submitted to assess the quality of life of the subjects and the ability to practice certain activities, including work and sports. Complications were evaluated according to the modified Clavien–Dindo–Sink classification; Results: The mean follow-up was 5 years (range, 1.0–13.6). Clavicular osteotomy, performed in 15 patients, improved post-operative abduction by a mean of 25° (95% CI: 11–39°; *p* = 0.001). Three patients had complications (7.1%), with two requiring re-operation. At follow-up, 67.5% of patients had a qDASH score < 7, highlighting excellent functional outcomes. Shoulder function showed moderate correlation with pre- and post-operative flexion. The SPADI and UCLASS scores indicated significant improvement, with 70.0% reporting high satisfaction. Factors like sex, associated anomalies, and surgical technique did not impact patient-reported outcomes or satisfaction; Conclusions: The modified Green’s technique has proven to be a safe procedure with a low rate of complications and satisfactory clinical and functional patient-reported outcomes.

## 1. Introduction

The congenital elevation of the scapula, or Sprengel’s Deformity (SD), is a rare congenital deformity of the shoulder girdle, characterized by an abnormal location of the scapula during development, leading to a hypoplastic, elevated and a malrotated scapula [1,2]. It represents the most common congenital deformity of the shoulder in children with a reported incidence of 0.3 cases per 10,000 live births [3,4]. SD is often found in association with other disorders such as Klippel–Feil syndrome and scoliosis [2,5]. It shows a female predominance, mostly a unilateral presentation, that more frequently involves the left side [2,5,6]. When symptomatic, the disorder varies in severity from a slightly limited shoulder range of motion (ROM) and mild cosmetic deformity to more pronounced dysfunction and severe clinical abnormalities [2]. Improving cosmetic appearance and addressing ROM limitations are common reasons for seeking treatment, but clear guidelines on indications for surgery and timing are still lacking. Various surgical techniques have been described for treating SD, among these, Green’s and Woodward’s procedures, along with their respective variants, are the most utilized procedures, each with respective variants, appearing as the most widely common [7,8,9,10]. A recent systematic review found that both techniques yield generally satisfactory outcomes, yet neither has shown clear superiority over the other [11]. While a common consensus exists on the effectiveness of surgery in enhancing scapular appearance and position, there remains a dearth of consistent data on post-operative patient-reported clinical and functional outcomes. In this context, Green’s technique for SD was reported as a viable surgical option, offering promising results [12]. In the literature, only two studies [9,13] reported patient-reported outcome measures (PROMs) in a cohort of patients who underwent surgery for SD with Woodward’s technique. To date, no study has assessed clinical and functional outcomes through PROMs in pediatric patients with SD treated with Green’s procedure. This study aims to present the combined experience of two pediatric orthopedic tertiary referral departments in managing SD using a modified Green’s procedure, with a particular focus on patient-reported clinical and functional outcomes. We hypothesized that most treated patients would attain excellent clinical–functional outcomes and a high level of satisfaction. Furthermore, we sought to identify any pre-operative and surgical factors that could have impacted patient-reported outcomes.

## 2. Materials and Methods

### 2.1. Study Design and Patient Selection

**Figure 1:**
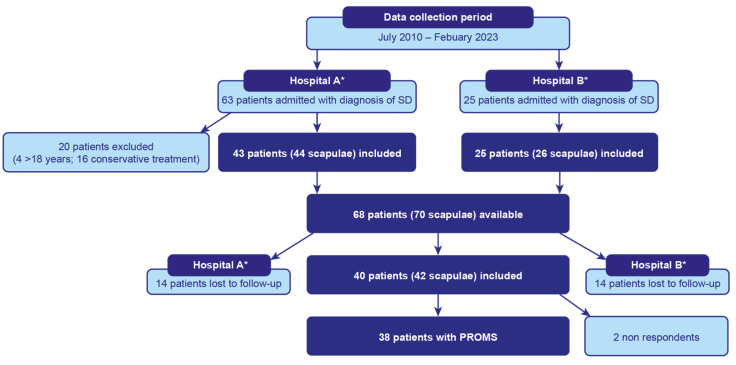
Flowchart illustrating the study design. * Patient data were extracted from the hospital’s electronic database using the ICD-9-CM code 755.52 (“congenital elevation of the scapula” or “Sprengel’s Deformity”).

This is a retrospective multicentric study with prospectively collected data investigating a cohort of consecutive children undergoing corrective surgery for SD at two tertiary referral institutions for paediatric orthopaedics. In accordance with the procedures and policies established by the Helsinki Declaration, ethics approval was obtained (n. 353/2021/Oss/IOR–22 April 2021), and caregivers gave informed consent for study participation. A search of electronic records in both hospitals identified all modified Green procedures performed from July 2010 to February 2023 using ICD-9-CM code 755.52 (congenital elevation of scapula or Sprengel deformity) (Figure 1). The exclusion criteria were: (1) an age over 18 years; (2) conservative cases. Charts and medical records were reviewed and investigated for demographics, clinically associated conditions, characteristics and ROM (in terms of abduction and flexion) of the affected shoulder, age at treatment, type of surgical technique, length of follow-up, rate of complications and recurrences. Shoulder abduction was measured with the patient supine at the edge of the bed. The fulcrum of the goniometer was placed on the anterior aspect of the acromion process. The stationary arm was aligned with the midline of the sternum, and the moving arm with the anterior midline of the humerus. The thorax was stabilized with one hand, while the wrist was grasped and the arm lifted with the other. Shoulder flexion was measured with the patient supine. The center of the goniometer was placed on the acromion process. The stationary arm was aligned with the lateral midline of the trunk, and the moving arm with the lateral midline of the humerus, toward the lateral epicondyle. The palm faced the body with the thumb pointing upward. The arm was then raised overhead, keeping the elbow as straight as possible.

**Figure 2:**
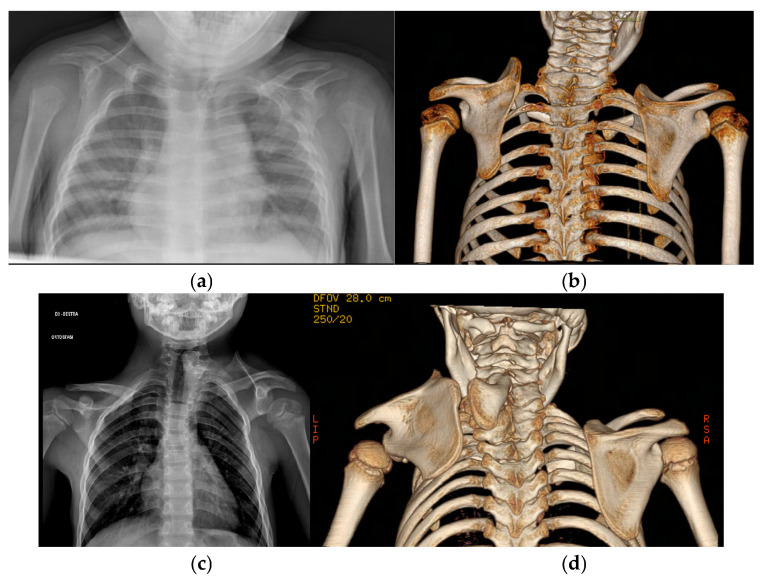
Radiographic imaging of Sprengel’s shoulder: (**a**) plain AP radiograph shows Type 2 SD according to Rigault with scapula apex at the C5 level; (**b**) the 3D CT scan shows elevation of the left scapula, with no vertebral connections; (**c**) plain AP radiograph shows Type 3 SD according to Rigault with scapula apex at the C4 level; (**d**) the 3D CT scan reveals the presence of an omovertebral bone and multiple cervical fusions on the right side, consistent with Klippel–Feil syndrome.

The Cavendish classification was utilized for clinical assessment, categorizing SD into four distinct grades: Grade 1: minimal deformity, shoulders are level, nearly invisible under clothing; Grade 2: mild deformity, visible bump, but glenohumeral joints remain level; Grade 3: moderate deformity with 2–5 cm elevation of the affected shoulder; Grade 4: severe deformity with >5 cm elevation, often accompanied by neck webbing [6]. Anteroposterior radiographs of both shoulders were assessed, and SD was graded using Rigault’s classification [14]. This system categorizes SD based on the position of the scapula’s superomedial angle relative to the adjacent vertebral level. In grade 1, the superomedial angle is below T1; in grade 2, it lies between T1 and C5; and in grade 3, it is positioned above C5 (Figure 2).

### 2.2. Surgical Technique

**Figure 3:**
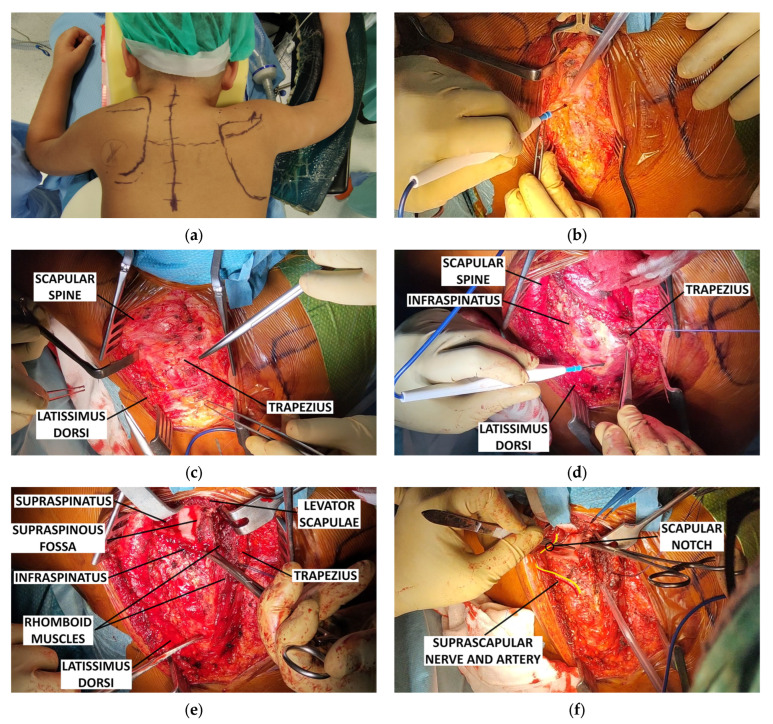

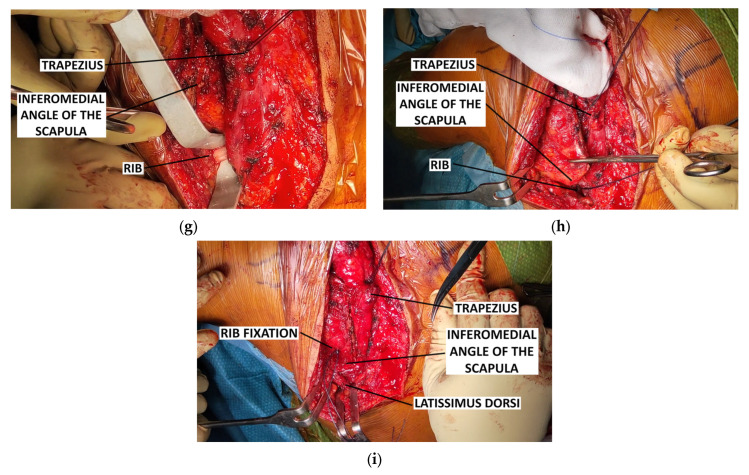
The steps of the surgical procedure: (**a**) cutaneous landmarks; (**b**) longitudinal incision and dissection of subcutaneous tissues; (**c**) identification of the trapezius; (**d**) the trapezius is detached from its scapular origin and reflected medially; (**e**) full exposure of the scapula and superomedial angle; (**f**) neurovascular bundle; (**g**) isolation of a lower rib; (**h**) caudal translation of the scapula; (**i**) fixation of the inferomedial angle of the scapula to the lower rib.

All procedures were performed by four pediatric orthopedic surgeons each with over 10 years of experience in pediatric upper limb surgery, including two from the European Pediatric Orthopedic Society (EPOS) Upper Limb Study Group. Patients underwent the modified Green’s procedure with minimal variations among surgeons, including skin incision site, inferior scapular apex anchorage, and clavicular osteotomy use. Under general anaesthesia, the patient was put in prone position, the landmarks of the elevated scapula and of the opposite side were identified and marked (the vertebral border, the inferior angle, and the scapular spine) as shown in Figure 3a. A longitudinal midline skin incision from the spinous process of the fourth cervical vertebra to the tenth thoracic vertebra (C4 to T10) or, in alternative, a more lateral incision along the medial border of the elevated scapula in a curvilinear fashion was made according to the surgeon preference (Figure 3b). After skin and subcutaneous dissection (Figure 3c), the insertion of the trapezius muscle on the scapular spine was sectioned and reflected medially to expose the underlying muscles (Figure 3d). The supraspinatus muscle was detached from the scapula and, if present, the omovertebral bone was excised. The insertion of the levator scapulae muscle and of the rhomboideus muscles were dissected, exposing the entire scapula, and the supraspinous part of the scapula was excised when redundant or hook shaped (Figure 3e). At this stage, it is crucial to take care to avoid injuring the neurovascular bundle (Figure 3f). After the removal of the fibrous bands that in some cases connected the scapula with the chest, the scapula was gently distally transferred and then rotated into the desired position. In some cases, the subscapularis and infraspinatus muscles were detached to improve scapular mobilization and then reattached in a more functionally optimal, superior position. After clinically checking the ROM’s improvement of the shoulder, the inferior angle of the scapula was placed in a muscular pocket created within the latissimus dorsi muscle and secured to a rib with absorbable sutures (Figure 3g–i). All muscles were reattached in an improved, normalized position after their lengthening if necessary. Standard layers closure was performed. In certain cases, a clavicle osteotomy was combined with the Green procedure as an initial step, depending on the surgeon’s experience and preference., to reduce the risk of brachial plexus palsy. In supine position, a supraclavicular linear incision was made 1 cm above the clavicle, centred over the clavicle midpoint, and extended for 4–5 cm. After protecting the neurovascular structures, with an oscillating electric saw, a single-level or double-level clavicle osteotomy was performed, maintaining the posteroinferior cortex. The periosteum and the skin over the clavicle were then closed with an absorbable suture. After surgery, the patient was protected with a Velpeau bandage for 3–4 weeks, then started a gradual rehabilitation program with active exercises, focusing on shoulder and upper limb movements, particularly flexion and adduction. We immediately encourage swimming and water activities. At the 3–6-month follow-up, depending on the patient’s condition, we recommend resuming unrestricted sports, including competitive contact and overhead sports. Due to the patient’s age and the rare condition, we do not follow a specific step-by-step rehabilitation program but encourage spontaneous active recovery of the full range of motion.

### 2.3. Follow-Up Assessment

Follow-up visits and tests were conducted by independent observers not involved in the initial surgery. At follow-up, we assessed the onset of complications or recurrence of the deformity, the cosmetic appearance, and the shoulder’s range of motion (ROM). Complications were collected in accordance with the modified Clavien–Dindo–Sink (CDS) classification [15,16]. Parents were contacted by phone, informed about the study, asked for consent, and sent the survey via email. All patients were invited to complete the quick Disability of the Arm, Shoulder and Hand scores (qDASH) [17], the Shoulder Pain Index and the Shoulder Disability Index (SPADI) [18] and the University of California at Los Angeles Shoulder Score (UCLASS) [19]. All questionnaires were administered in their translated versions in the official languages of the participating hospitals [18,20,21,22]. The qDASH is validated in pediatric patients over 8 years old with good responsiveness [17,23]. Scores range from 0 (no disability) to 100 (maximum disability), with higher scores indicating greater disability [17]. In our study, we excluded the work module due to patient age and used normative data for young adults since baseline Quick-DASH scores were not collected. A score of ≤7 was considered within the first quartile of healthy individuals, while sports module scores of 6 for males and 13 for females were also within this range [24]. Scores below these thresholds suggest no perceived clinical or functional issues with the affected elbow. The Shoulder Pain and Disability Index (SPADI) is a patient-completed questionnaire with 13 items assessing pain levels and difficulty with ADLs requiring the use of the upper extremities. The pain subscale (Shoulder Pain Index: SPI) includes 5 items, while the disability subscale (Shoulder Disability Index: SDI) includes 8 items. The pain scale totals 50, and the disability scale totals 80. The overall SPADI score is expressed as a percentage, with 0 indicating the best outcome and 100 the worst. Higher scores indicate greater disability. To date, no threshold values or normative scores from the general population have been reported to evaluate the quality of clinical outcomes.

For UCLASS, a threshold of 30 indicated successful treatment, reflecting both complete functional recovery and satisfaction with surgical outcomes. Patients scoring above this threshold were considered to have achieved a successful outcome [25]. Overall patient satisfaction with both functional outcomes and aesthetic results was assessed using an 11-point Numeric Rating Scale (NRS), where 0 indicated extreme dissatisfaction and 10 indicated extreme satisfaction. Scores were then converted to a five-point Likert scale, with interpretations as follows: 0–2, extremely dissatisfied; 3–4, dissatisfied; 5–6, neutral; 7–8, moderately satisfied; 9–10, extremely satisfied [26].

### 2.4. Statistical Analyses

Data were entered into an Excel spreadsheet with patient-assigned numerical codes. Categorical variables are presented as raw numbers and proportions with 95% confidence intervals (95% CI), while continuous data are expressed as mean ± standard deviation (SD) and range. Normality was assessed using the Kolmogorov–Smirnov test, with non-parametric statistics applied to non-normally distributed data. Group comparisons were performed using the student’s *t*-test or Mann–Whitney U-test for continuous variables, depending on data distribution, and Fisher’s exact test for categorical variables. Pre-operative and post-operative comparisons were analysed using the paired Student’s *t*-test for normally distributed data or the Wilcoxon signed-rank test for non-normally distributed data. Bivariate analyses were conducted using Pearson or Spearman correlation coefficients, selected based on the distribution of the variables. Correlation coefficients were interpreted as negligible or none (<0.10), weak (0.10–0.39), moderate (0.40–0.69), strong (0.70–0.89), and very strong or perfect (0.90–1.00). Exploratory univariable and multivariable analyses were conducted using linear or logistic regression, as appropriate, to identify potential associations between baseline variables and outcomes. Linear mixed effect models with patient as random effect were used, to avoid violation of the principle of independence in bilateral cases. Statistical significance was set at *p* < 0.05. All analyses were conducted using SPSS (version 25.0; IBM Corp, Armonk, NY, USA).

## 3. Results

### 3.1. Patient Demographics and Baseline Characteristics

During the study, 68 children underwent Green’s procedure for SD. Twenty-eight were excluded due to loss to follow-up, leaving 40 children for analysis (17 females, mean age at surgery: 7.3 ± 4.2 years; two bilateral cases). Patients’ demographics and clinical characteristics at baseline are reported in Table 1. Almost two-thirds of the patients had associated anomalies, with scoliosis being the most common. Additionally, Klippel–Feil syndrome was reported in 15 patients, including both bilateral cases. The scapula was hypoplastic in 29 cases (69.0%), with an omovertebral bone bar present in 19 cases (45.2%). A detailed description of the scapula’s clinical appearance according to the Cavendish classification was reported in 21 patients, with 90% classified as grade 3 or 4. Pre-operative shoulder abduction and flexion averaged 77° ± 26° (range 10–105°) and 96° ± 32° (range 22–157°), respectively. Radiographs showed 61.9% of cases as Rigault grade 2 and 26.2% as grade 3. The Rigault classification correlated moderately with both the pre-operative Cavendish score (Spearman’s r = 0.61, *p* = 0.006) and shoulder abduction (Spearman’s r = −0.43, *p* = 0.007).

### 3.2. Surgical Variable Complications and Outcomes

**Figure 4:**
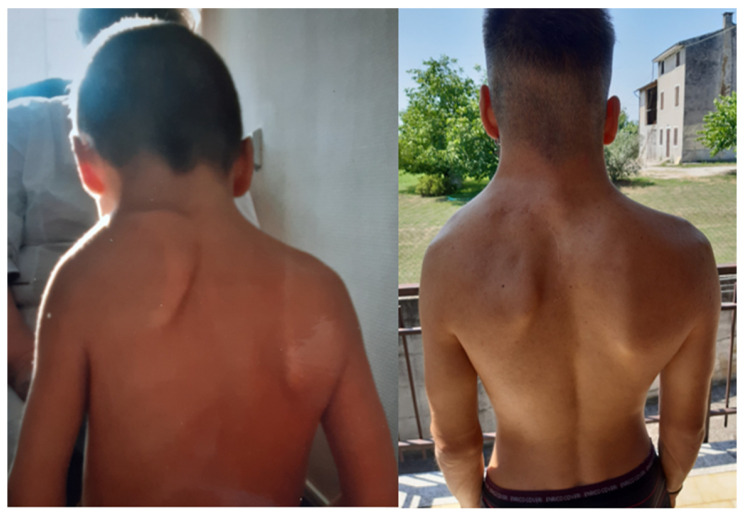
Patient with SD and congenital muscular torticollis at 8-year follow-up.

**Figure 5:**
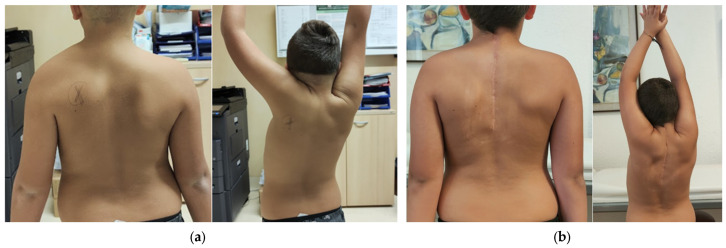
Clinical evaluation at the baseline and at 6-month follow-up: (**a**) pre-operative ROM; (**b**) post-operative clinical evaluation.

All patients were surgically treated to place the scapula in a more physiological position. The incision was midline longitudinal in 29 cases and curvilinear centered on the medial scapular border in the rest. Redundant medial scapular apex resection was performed in 19 cases. Scapula fixation involved rib attachment in 29 cases and a latissimus dorsi muscular pocket in the others. Clavicular osteotomy was performed in 15 patients (35.7%). These patients had lower pre-operative abduction (mean difference 14.8°; *p* = 0.017). Clavicular osteotomy was associated with increased post-operative abduction (Mean Difference 25°; 95% CI: 11–39°: Mann–Whitney U-test, *p* = 0.001) without added morbidity or complications. Following surgery, clinical appearance improved to grade 1 in 85.3% of cases (Figure 4) and to grade 2 in 14.7%, according to the Cavendish classification (*p* < 0.0005). Both shoulder abduction (Figure 5) and flexion showed significant improvements of 60° ± 24° and 47° ± 30°, respectively (*p* < 0.0005). Three patients experienced complications, two of which were classified as major. One involved a vertebral–scapular bone bridge causing cervicalgia, and another had heterotopic ossification; both required re-operation. The third patient experienced a partial recurrence of scapular cranialization but no further surgical intervention was needed due to the satisfactory functional outcome. The post-operative clinical features are summarized in Table 2.

### 3.3. Follow-Up

The mean follow-up period was 5 years (range: 1–11.2 years), and the mean age at the survey was 12.6 ± 5 (range 5–23) years. Thirty-eight out of forty patients completed and returned the survey questionnaires. All examined scores showed moderate to strong correlations with each other (Spearman’s r from 0.53 to 0.77). At follow-up, the mean qDASH total score was 11.5 ± 15.2 points (range: 0–50), while the sport module score averaged 8.1 ± 14.7 (range: 0–62.5). Sixty-seven percent of patients reported a qDASH score below 7 points, which falls within the normal range for the general population. qDASH showed a poor correlation with the pre-operative abduction (Spearman’s r = −0.32; *p* = 0.041) and the Rigault score (Spearman’s r = 0.36; *p* = 0.030), but a moderate correlation with both pre-operative (Spearman’s r = −0.53; *p* = 0.001) and post-operative (Spearman’s r = −0.64; *p* < 0.0005) shoulder flexion and abduction (Spearman’s r = −0.42; *p* = 0.008), a relationship also confirmed in the qDASH sport module and in the other PROMs. The total SPADI score was 15.6 ± 22.3 points (0–90) (mean Shoulder Pain Index: 12.5 ± 21.9, mean Shoulder Disability Index: 17.3 ± 22.7). The Shoulder Pain Index showed a poor correlation with age at surgery (Spearman’s r = 0.36; *p* = 0.024) but a moderate correlation with age at follow-up (Spearman’s r = 0.43; *p* = 0.005), pre-operative Cavendish score (Spearman’s r = 0.45; *p* = 0.041) and post-operative flexion (Spearman’s r = −0.45; *p* < 0.003), the latter also correlating with the Shoulder Disability Index (Spearman’s r = −0.46; *p* = 0.003). The UCLASS was 31.5 ± 5.1 points (range 16–35) and showed poor-to-moderate correlation with age at follow-up (Spearman’s r = −0.40; *p* = 0.011), pre-operative abduction (Spearman’s r = 0.43; *p* = 0.006) and flexion (Spearman’s r = 0.53; *p* = 0.001) and post-operative abduction (Spearman’s r = 0.49; *p* = 0.002) and flexion (Spearman’s r = 0.56; *p* < 0.0005). Thirty-one patients (77.5%) reported a UCLASS score of 30 points or more. The mean grade of satisfaction was 8.3 out of 10 for the functional aspects and 7.8 out of 10 for the cosmetic issue. Among the respondents, 29 patients (72.5%) participated in sports or played musical instruments. Of these, 22 were involved in upper limb-intensive sports such as basketball, volleyball, swimming, tennis, and karate. No statistically significant differences in age, shoulder mobility, pain, or functionality were found compared to those not involved in these activities. Overall, 30 patients were very satisfied with the functional outcome (≥8/10), and 31 patients were very satisfied with the aesthetic outcome (≥8/10). Based on the available data, factors such as sex, side, associated anomalies (including scoliosis and Klippel–Feil syndrome), and surgical technique did not impact PROMs or patient satisfaction regarding functional ability and aesthetic appearance.

## 4. Discussion

This study presents clinical and radiographic data, along with patient-reported outcomes, from one of the largest case series in the literature on the surgical treatment of SD using the modified Green technique. Our findings confirm that the modified Green procedure is safe, with a low complication rate, and effectively increases shoulder range of motion by approximately 47° to 60°. Furthermore, it enhances functionality and improves the aesthetic appearance of the neck contour.

In our cohort, we observed a predominance of the left-side involvement (54.8%) and a slightly higher male prevalence (57.5%) [11]. We identified 19 cases (47.5%) with congenital scoliosis and 15 cases (37.5%) with Klippel–Feil syndrome. Additionally, 29 patients (72.5%) had other associated conditions, including rib and vertebral anomalies, torticollis, kidney diseases, and multiorgan malformations. Nearly half of our patients (45.2%) had an abnormal vertebral bone connection, and we found a slightly higher incidence of unilateral kidney (6.8%) compared to the literature (2.5%). The Rigault classification demonstrated a moderate correlation between the shoulder profile and abduction, aligning with Vuillermin et al. [27]. This radiographic classification proves useful for predicting clinical outcomes and the need for surgery.

Regarding the surgical technique, there is still no consensus on the necessity of performing clavicle osteotomy as an adjunctive procedure. Some surgeons perform it in all cases, while others never do, and some decide based on patient characteristics such as age, degree of adduction, or initial clinical appearance. However, to our knowledge, no studies have reported statistically significant clinical differences between patients who undergo clavicle osteotomy and those who do not. In our case series, we found that clavicle osteotomy—performed based on the surgeon’s experience and preference—was predominantly carried out in children with lower pre-operative abduction to reduce the risk of brachial plexus palsy. In fact, this adjunctive surgical procedure can enhance shoulder abduction without adding significant surgical risks. Aslani et al. [28] reported on 31 patients treated with vertical osteotomy for SD, noting that 19 underwent concurrent clavicle osteotomy while 12 did not, without comparing the outcomes between the groups. Similarly, Naik [29] described 68 patients treated with the Green technique, including 34 who had clavicle osteotomy, but did not explore clinical differences between the groups. Nonetheless, they recommend this additional procedure for patients with a Cavendish score greater than 2. Conversely, we found no differences in clinical and functional outcomes related to the type of incision and fixation. However, some authors suggest that fixation within the latissimus dorsi pocket rather than to a rib may yield better clinical results.

Indications for the surgical treatment of SD are reported as grades 3 and 4 according to the Cavendish classification, as grades 2 and 3 according to Rigault’s classification and in case of considerable functional limitations of the ROM [6,30,31]. Many surgical techniques with satisfactory results have been described for SD, but an evaluation of the different results is nearly impossible because of the procedures authors’ modifications and the rarity of the condition. As recently reported, Green’s and Woodward’s techniques (with their modified variants) cover 70% of all surgical procedures [11]. In addition, the best clinical and cosmetic results seem to be achieved when surgery is performed in children aged less than eight years [11]. For a variety of reasons, we preferred the modified Green’s procedure. First, this technique permits better visualization of the superomedial part of the scapula that can be easily excised when redundant; secondary, acting on a malformed scapula could appear easier than on a dysmorphic vertebra. Moreover, muscular realignment to improve dynamic vectors is easier in this technique. On the contrary, other techniques, such as Woodward’s procedure, avoid the stretching and the lengthening of the muscles, that are not excised from the scapula.

In this study, we decided to analyze only the surgical cases treated because of the functional limitation or the cosmetic deformity correlated to the disease. The mean age at treatment was 7.3 years and all cases were treated according to the modified Green’s procedure with the associated omovertebral bone resection, if present. There were only three complications, with two cases requiring surgical reoperation. Overall, these data confirm that the Green procedure is safe.

We analyzed the pre-operative and post-operative ROM in terms of abduction and flexion. An increase in the post-operative ROM was found, showing a clear improvement in daily life activities. Moreover, pre-operative ROM, including shoulder abduction and flexion, is a significant predictor of post-operative outcomes. Children with reduced shoulder mobility at baseline tend to have slightly poorer clinical and functional outcomes post-operatively and over time.

No prior studies have used validated patient-reported outcome tools for patients treated with Green’s technique for SD. Only two studies have assessed patient-reported outcomes for the Woodward procedure. Ashok [9] and Walstra [13] had previously reported validated scores (qDASH, SST, PODCI [32]) for the post-operative assessment of their cohort. Walstra et al. found that eight children achieved a post-operative Constant Score of 85 points, a DASH score of 14.59, and an SST score of 9.5. Similarly, Ashok reported an SST score of 9.6 points and a post-operative PODCI score of 24.07 in 14 children who received surgery for SD. In both cases, patients were treated according to Woodward’s procedure. In our opinion, a global and detailed clinical evaluation of patients is highly recommended because of the severe functional limitations that SD could determine also in daily life basic activities. For this reason, we submitted several clinical scores to our surgical patients to evaluate their grade of satisfaction after treatment and their ability in the various spheres of daily life. Submitting the qDASH (with the sport module), SPADI (disability index and pain index) and UCLA Shoulder Score, we reported good results in all cases and a high level of satisfaction. Concerning sports, 76.9% of patients preferred to practice sports activities; among them, 19 patients (63.3%) performed a physical activity in which the upper limb is particularly involved (basketball, volleyball, tennis, karate, swimming, rhythmic gymnastics). Although satisfactory results of the submitted scores, from our analysis, we found a negative trend in almost all of them in relation to the extended follow-up period. Since the low number of patients, a statistical analysis does not appear adequate, but this phenomenon appeared interesting and worthy of further consideration.

In our cohort, age at treatment did not significantly influence clinical and functional post-operative outcomes, likely because patients underwent surgery before age 8, as suggested by the recent literature. Only the Shoulder Pain Index showed a slight direct correlation with age at surgery, suggesting older patients may experience more residual pain. Additionally, age at follow-up had a mild to moderate correlation with the Shoulder Pain Index and UCLASS, indicating increased shoulder pain in adulthood. This suggests that extended rehabilitation or physical therapy may be beneficial for preventing long-term pain and possible functional decrease after surgery.

From our analysis, we would highlight a correlation between pre-operative shoulder flexion and post-operative outcomes. As Dayanidhi et al. [33] suggested, unlike adults, children receive substantial contribution from scapular external rotation during shoulder flexion. Soldado et al. [34] studied 23 patients with SD, finding that more severe deformity and reduced flexion were associated with inferior glenohumeral stiffness, measured by the spino-humeral abduction angle. Thus, reduced flexion was not only due to scapulothoracic muscle weakness but also to the rigidity of the inferior capsule. Internal rotation of the scapular inferior pole, scapulothoracic muscle weakness, and capsular contracture negatively affect the shoulder’s range of motion, especially in terms of shoulder flexion. The finding of a marked limitation in terms of pre-operative flexion can, therefore, be regarded as a negative prognostic factor. This knowledge, however, could be useful in focusing the post-operative physiotherapeutic treatment.

The present study has limitations. The retrospective nature of the study and the small number of cases limit our analysis and their statistical strength. In some cases, the pre-operative and post-operative characteristics appeared incomplete and not always reported in our records, as Cavendish’s and Rigault’s classification and the measure of the pre-operative and post-operative ROM. The involvement of four surgeons across two hospitals may have introduced additional biases, beyond the identified technical variations, affecting the results. With the available sample size, we could only demonstrate the effect of the additional clavicular osteotomy, though other surgical variables may also influence the outcome. Patients were contacted by phone or email, and the questionnaires were completed remotely. This means that the parent with access to the email and who provided consent may have filled out the forms instead of the patient. This raises concerns about the reliability of the results, as potential discrepancies between parent and child responses are unknown. Furthermore, the scores we submitted are not specific for pediatric patients and a more appropriate evaluation would be necessary. Finally, a comparison of such scores by submitting them also in the pre-operative evaluation could permit us to reach more adequate results with statistical validity. For these reasons, a prospective study could be necessary to better evaluate the improvement referred to by the patient himself.

## 5. Conclusions

To the best of our knowledge, this paper represents one of the largest studies on SD cases treated with Green’s procedure. We focused on post-operative results using various validated questionnaires and found the procedure to be safe, with a low complication rate and satisfactory outcomes. Pre-operative adduction and flexion are significant predictors of post-operative outcomes. While additional clavicle osteotomy can increase shoulder abduction by an average of 25°, it does not significantly impact clinical and functional outcomes. Clinical and functional results tend to deteriorate slightly over time, especially regarding residual pain. Therefore, long-term monitoring and additional therapeutic strategies are recommended to address this issue.

## Figures and Tables

**Table 1:** Patients’ demographics and clinical characteristics at baseline.

		Total
Patients (shoulders)		40 (42)
Bilateral cases		2 (5.0%)
Sex	Male	23 (57.5%)
	Female	17 (42.5%)
Associated conditions	Scoliosis Klippel–Feil syndrome Others	19 (47.5%) 15 (37.5%) 29 (72.5%)
Side	Right	19 (45.2%)
	Left	23 (54.8%)
	Unreported	0 (0%)
Scapular features	Hypoplastic Normal Unreported	29 (69.0%) 7 (16.7%) 6 (14.3%)
Vertebral connection	Omovertebral bone Fibrous band None Unreported	19 (45.2%) 3 (7.2%) 19 (45.2%) 1 (2.4%)
Pre-operative Rigault score	0 1 2 3 Unreported	0 (0%) 1 (2.4%) 26 (61.9%) 11 (26.2%) 4 (9.5%)
Pre-operative Cavendish score	0 1 2 3 4 Unreported	0 (0%) 0 (0%) 2 (4.8%) 13 (30.9%) 6 (14.3%) 21 (50.0%)
Pre-operative ROM (abduction)	<20° 20–45° 45–90° 90–120° >120°	2 (4.8%) 2 (4.8%) 22 (52.4%) 16 (38.1%) 0 (0%)
Pre-operative ROM (flexion)	<45° 45–90° 90–135° >135° Unreported	3 (7.1%) 10 (23.8%) 23 (54.7%) 2 (4.8%) 4 (9.5%)

**Table 2:** Patients’ post-operative features.

		Total
Mean age at treatment	(years)	7.3 ± 4.2
Mean follow-up	(months)	59.0 ± 37.5
Associated clavicle osteotomy		15 (35.7%)
Complications		3 (7.1%)
Post-operative Cavendish	0 1 2 3 4 Unreported	0 (0%) 29 (69.0%) 5 (11.9%) 0 (0%) 0 (0%) 8 (19.0%)
Post-operative ROM (abduction)	<20° 20–45° 45–90° 90–120° >120° Unreported	1 (2.4%) 0 (0%) 12 (28.6%) 9 (21.4%) 18 (42.8%) 2 (4.8%)
Post-operative ROM (flexion)	<45° 45–90° 90–135° >135° Unreported	1 (2.4%) 1 (2.4%) 7 (16.7%) 31 (73.8%) 2 (4.8%)

## Data Availability

The data presented in this study are available upon request from the corresponding author due to patients’ privacy.

## References

[1] Grogan D.P., Stanley E.A., Bobechko W.P. (1983). The congenital undescended scapula. Surgical correction by the Woodward procedure. J. Bone Jt. Surg.-Ser. B.

[2] Harvey E.J., Bernstein M., Desy N.M., Saran N., Ouellet J.A. (2012). Sprengel deformity: Pathogenesis and management. J. Am. Acad. Orthop. Surg..

[3] Kim J.K., Cho T.J., Lee K., Moon H.J., Park M.S., Yoo W.J., Chung C.Y., Choi I.H. (2012). Atlantoaxial rotatory subluxation after surgical relocation of Sprengel deformity: A case report. J. Pediatr. Orthop. Part B.

[4] Satsuma S., Yamamoto T., Kobayashi D., Yoshiya S., Marui T., Akisue T., Hitora T., Nagira K., Ohta R., Kurosaka M. (2003). Extraabdominal desmoid tumor in a surgical scar of a patient with Sprengel’s deformity. J. Pediatr. Surg..

[5] Eulenberg M. (1863). Beitrag zue Dislocation der Scapula. Amtliche Berichte uber die Versammlungen Deutscher Naturforscher und Aerzte fur die Jahre.

[6] Cavendish M. (1972). Congenital elevation of the scapula. J. Bone Jt. Surg Br..

[7] Alsiddiky A.M., Rafiq Z., Bakarman K.A., Alhuzaimi F.S., Asif M. (2020). A Novel Modification of Woodward Procedure for Correction of Sprengel Deformity by Application of Anchoring Sutures. Indian J. Orthop..

[8] Andrault G., Salmeron F., Laville J.M. (2009). Green’s surgical procedure in Sprengel’s deformity: Cosmetic and functional results. Orthop. Traumatol. Surg. Res..

[9] Ashok A., James D., Gahukamble A., Palocaren T., Madhuri V. (2021). Modified Woodward’s procedure confers functional improvement in Sprengel’s deformity. J. Pediatr. Orthop. B.

[10] Wada A., Nakamura T., Fujii T., Takamura K., Yanagida H., Yamaguchi T., Kubota H., Oketani Y. (2014). Sprengel deformity: Morphometric assessment and surgical treatment by the modified green procedure. J. Pediatr. Orthop..

[11] Zarantonello P., Di Gennaro G.L., Todisco M., Cataldi P., Stallone S., Evangelista A., Ferrari D., Antonioli D., Trisolino G. (2021). Surgical treatment of Sprengel’s deformity: A systematic review and meta-analysis. Children.

[12] Gonen E., Simsek U., Solak S., Bektaser B., Ates Y., Aydin E. (2010). Long-term results of modified Green method in Sprengel’s deformity. J. Child. Orthop..

[13] Walstra F.E., Alta T.D., van der Eijken J.W., Willems W.J., Ham S.J. (2013). Long-term follow-up of Sprengel’s deformity treated with the Woodward procedure. J. Shoulder Elb. Surg..

[14] Rigault P., Pouliquen J., Guyonvarch G., Zujovic J. (1976). Congenital elevation of the scapula in children: Anatomopathological and therapeutic study apropos of 27 cases [French]. Rev. Chir. Orthop. Reparatrice Appar. Mot..

[15] Dindo D., Demartines N., Clavien P.A. (2004). Classification of surgical complications: A new proposal with evaluation in a cohort of 6336 patients and results of a survey. Ann. Surg..

[16] Dodwell E.R., Pathy R., Widmann R.F., Green D.W., Scher D.M., Blanco J.S., Doyle S.M., Daluiski A., Sink E.L. (2018). Reliability of the Modified Clavien-Dindo-Sink Complication Classification System in Pediatric Orthopaedic Surgery. JBJS Open Access.

[17] Quatman-Yates C.C., Gupta R., Paterno M.V., Schmitt L.C., Quatman C.E., Ittenbach R.F. (2013). Internal consistency and validity of the QuickDASH instrument for upper extremity injuries in older children. J. Pediatr. Orthop..

[18] Angst F., Goldhahn J., Pap G., Mannion A.F., Roach K.E., Siebertz D., Drerup S., Schwyzer H.K., Simmen B.R. (2007). Cross-cultural adaptation, reliability and validity of the German Shoulder Pain and Disability Index (SPADI). Rheumatology.

[19] Smith M.V., Calfee R.P., Baumgarten K.M., Brophy R.H., Wright R.W. (2012). Upper Extremity-Specific Measures of Disability and Outcomes in Orthopaedic Surgery. JBJS.

[20] Padua R., Padua L., Ceccarelli E., Romanini E., Zanoli G., Amadio P.C., Campi A. (2003). Italian version of the disability of the arm, shoulder and hand (DASH) questionnaire. Cross-cultural adaptation and validation. J. Hand Surg. Am..

[21] Huber W., Hofstaetter J.G., Hanslik-Schnabel B., Posch M., Wurnig C. (2004). The German version of the Oxford Shoulder Score--cross-cultural adaptation and validation. Arch. Orthop. Trauma Surg..

[22] Marchese G.S.C., Cristalli G., Pichi B., Manciocco V., Mercante G., Pellini R., Marchesi P., Sperduti I., Ruscito P. (2012). Italian cross-cultural adaptation and validation of three different scales for the evaluation of shoulder pain and dysfunction after neck dissection: University of California—Los Angeles (UCLA) Shoulder Scale, Shoulder Pain and Disability Index (SPADI). Acta Otorhinolaryngol. Ital..

[23] Kämppä N., Hulkkonen S., Grahn P., Laaksonen T., Repo J. (2024). The construct validity and internal consistency of QuickDASH in pediatric patients with upper extremity fractures. Acta Orthop..

[24] Aasheim T., Finsen V. (2014). The DASH and the QuickDASH instruments. Normative values in the general population in Norway. J. Hand Surg. Eur. Vol..

[25] Xu S., Chen J.Y., Lie H.M.E., Hao Y., Lie D.T.T. (2019). Determination of Threshold Scores for Treatment Success After Arthroscopic Rotator Cuff Repair Using Oxford, Constant, and University of California, Los Angeles Shoulder Scores. Arthroscopy.

[26] Puzzitiello R.N., Nwachukwu B.U., Agarwalla A., Cvetanovich G.L., Chahla J., Romeo A.A., Verma N.N., Forsythe B. (2020). Patient Satisfaction After Total Shoulder Arthroplasty. Orthopedics.

[27] Vuillermin C., Wang K.K., Williams K.A., Hresko M.T., Waters P.M. (2021). Sprengel’s deformity: An analysis of surgically and nonsurgically treated patients. J. Shoulder Elb. Surg..

[28] Aslani M.A., Mirzaee F., Baradaran A.F., Ziaei M.E., Zafarani Z., Aslani H. (2020). Results of surgical treatment for Sprengle’s deformity with vertical corrective scapular osteotomy. Ann. Med. Surg..

[29] Naik P., Chauhan H. (2020). Functional improvement in patients with Sprengel’s deformity following Modified Green’s procedure and simplified clavicle osteotomy—A study of forty cases. Int. Orthop..

[30] Farsetti P., Weinstein S.L., Caterini R., De Maio F., Ippolito E. (2003). Sprengel ’ s deformity: Long-term follow-up study of 22 cases. J. Pediatr. Orthop. B.

[31] Herring J. (2014). Tachdjian’s Pediatric Orthapaedics: From the Texas Scottish Rite Hospital for Children.

[32] Daltroy L.H., Liang M.H., Fossel A.H., Goldberg M.J. (1998). The POSNA pediatric musculoskeletal functional health questionnaire: Report on reliability, validity, and sensitivity to change. Pediatric Outcomes Instrument Development Group. Pediatric Orthopaedic Society of North America. J. Pediatr. Orthop..

[33] Dayanidhi S., Orlin M., Kozin S., Duff S., Karduna A. (2005). Scapular kinematics during humeral elevation in adults and children. Clin. Biomech..

[34] Soldado F., Di-Felice-Ardente P., Barrera-Ochoa S., Diaz-Gallardo P., Bergua-Domingo J.M., Knörr J. (2020). Passive range of glenohumeral motion in children with a Sprengel’s deformity. JSES Int..

